# Different associations between obesity and impaired fasting glucose depending on serum gamma-glutamyltransferase levels within normal range: a cross-sectional study

**DOI:** 10.1186/1472-6823-14-57

**Published:** 2014-07-12

**Authors:** Nam Soo Hong, Jeong-Gook Kim, Yu-Mi Lee, Hyun-Woo Kim, Sin Kam, Keon-Yeop Kim, Ki-Su Kim, Duk-Hee Lee

**Affiliations:** 1 Department of Preventative Medicine, School of Medicine, Kyungpook National University, 680 Gukchaebosang-ro, Jung-gu, Daegu, South Korea; 2 Department of Life Science, Gachon University, 191 Hambakmoero, Yeonsu-gu, Incheon, South Korea; 3 Department of Family Medicine, Daegu Medical Center, 157 Pyungli-ro, Seo-Gu, Daegu, South Korea; 4 BK21 Plus KNU Biomedical Convergence Program, Department of Biomedical Science, Kyungpook National University, 680 Gukchaebosang-ro, Jung-gu, Daegu, South Korea

**Keywords:** γ-Glutamyltransferase (GGT), Impaired fasting glucose, Obesity, Type 2 diabetes

## Abstract

**Background:**

Despite the consistent relationship between serum γ-glutamyltransferase (GGT) and type 2 diabetes (T2D), one unsolved issue is the role of serum GGT in the well-known association between obesity and T2D. This study was performed to investigate whether the association between body mass index (BMI) and impaired fasting glucose (IFG) differed depending on serum GGT levels within the normal range.

**Methods:**

Study subjects were 2,424 men and 3,652 women aged ≥ 40, participating in the Fifth Korean National Health and Nutrition Examination Survey. Serum GGT levels within the normal range were classified into gender-specific tertiles.

**Results:**

Among men and women belonging to the lowest tertile of serum GGT, BMI showed statistically non-significant weak associations with the risk of IFG. However, among persons in the highest tertile of serum GGT, the risk of IFG was 3 − 4 times higher among persons with BMI ≥ 25 kg/m^2^ than those with BMI < 23 kg/m^2^ (P_interaction_ = 0.032 in men and 0.059 in women).

**Conclusions:**

The well-known strong association between BMI and IFG was observed mainly among persons with elevation of serum GGT to certain physiological levels, suggesting a critical role of serum GGT in the pathogenesis of IFG. This finding has an important clinical implication because serum GGT can be used to detect high-risk obese persons.

## Background

Serum γ-glutamyltransferase (GGT) within the normal range has emerged as an important predictor of type 2 diabetes (T2D) among various populations [1-5]. However, the role of serum GGT in the well-known association between obesity and T2D is still unclear. Some epidemiological studies have demonstrated statistically significant and borderline significant interactions between serum GGT and obesity in relation to the risk of T2D [6-8]. Importantly, even little association between obesity and T2D among persons in the very low normal range of serum GGT were reported in previous studies [7,8]. These findings suggest that the elevation of serum GGT to certain physiological levels is a prerequisite condition for obesity to increase the risk of T2D.

However, the findings on the interactions between serum GGT and obesity from previous epidemiological studies were not consistent; some studies failed to reach statistical significance with multiplicative interaction terms [3,5,9,10]. In addition, when gender-specific analyses were performed, the meaningful interactions were demonstrated among women only [6,8]. Therefore, further studies are required to investigate the possible interaction between serum GGT and obesity associated with the risk of T2D.

In this study, we hypothesized that if there truly were interactions between serum GGT and obesity in relation to the risk of T2D, the pattern might be observed more clearly among individuals with prediabetes. As patients with T2D are generally advised to lose weight [11] and serum GGT is also related to a change in body weight [12], the results could be diluted or distorted with T2D as the primary outcome of interest. Thus, this study was performed to investigate whether there were interactions between serum GGT within the normal range and obesity in association with the risk of impaired fasting glucose (IFG), especially focusing on the possibility of little relationship between obesity and IFG among persons with a very low normal range of serum GGT, after excluding patients with T2D.

## Methods

### Study population

This study analyzed data from the Fifth Korea National Health and Nutrition Examination Survey (KNHANES V) conducted by the Korea Centers for Disease Control and Prevention (KCDC) from 2010 − 2011. KNHANES V used stratified, multistage clustered sampling in order to collect a sample representing the Korean population. After we stratified the population first according to province and then to types of houses, we extracted 192 primary sampling units. Among the extracted sampling units, we extracted 20 houses per each sampling unit by systematic sampling. Specifically, 8,958 subjects were included in 2010 with a participation rate of 81.9%, and 8,518 subjects were included in 2011 with a participation rate of 80.4% [13]. Among 7,017 subjects who were over 40 years old with normal serum GGT (serum GGT < 73 U/L in men and < 48 U/L in women) [14] and with information on diabetic status, we excluded persons with physician-diagnosed diabetes (including the use of diabetic medication) (n = 780), fasting blood glucose ≥ 126 mg/dL (n = 551), or missing information on BMI (n = 15). The final sample sizes were 2,424 men and 3,652 women. This study was reviewed and approved by the Institutional Review Board of KCDC (IRB No. 2010-02CON-21-C, 2011-02CON-06-C), and written informed consent was obtained from all subjects.

### Measurements

KNHANES V consisted of a health interview survey, a health examination survey, and a nutrition survey. The data for the health interview and nutrition surveys were collected through individual interviews. Each participant’s serum was collected after overnight fasting. The samples were transported to the core laboratory and analyzed within 24 hours after collection. Serum glucose and serum GGT were analyzed using the Hitachi 7600 analyzer. Height was measured in units of 1 millimeter (mm), and body weight was measured in units of 0.1 kg using an automatic instrument.

### Statistical analysis

KNHANES V was designed as a complex sample, and data analysis considering stratification, cluster, and weight was employed. In this study, we defined IFG as fasting serum glucose between 100 mg/dL and 126 mg/dL. BMI was classified into three categories (<23, 23–25, and > 25 kg/m^2^), and serum GGT was categorized into gender-specific tertiles. Cutoff points were 22 U/L and 34 U/L in men and 14 U/L and 19 U/L in women. Rather than use continuous forms of BMI and serum GGT, we elected to categorize these variables to make the interpretation of results easier to comprehend and compare to previous research.

First, we examined the associations of IFG with serum GGT or BMI, not considering the possible interaction between GGT and BMI. Next, we analyzed the relationship between BMI and IFG after stratification by serum GGT into gender-specific tertiles. Analyses were adjusted for age, alcohol consumption (daily alcohol intake amount), smoking status (current smoker, former smoker, or never smoker), pack-years of cigarette smoking, and physical activity (frequency of days with moderate or vigorous exercise during the previous week). To evaluate the possible interaction between BMI and serum GGT, the multiplicative interaction term of the three categories of BMI and the gender-specific tertiles of serum GGT was included in the multiple logistic regression models. SAS version 9.3 (SAS, Inc., Cary, NC, USA) was used for all statistical analyses.

## Results

The general characteristics of the study subjects are shown in Table 1. Men and women with high normal serum GGT were more obese and included more current smokers and more current drinkers.

**Table 1:** General characteristics of study participants by γ-glutamyltransferase (GGT) tertiles

	**Tertiles of serum GGT**			**p**
	**Tertile 1 (0 ~ 22 U/L)**	**Tertile 2 (23 ~ 34 U/L)**	**Tertile 3 (35 ~ 73 U/L)**	
**Men (n = 2424)**	N = 822	N = 800	N = 802	
Age (years)	56.2 ± 0.5	54.0 ± 0.5	52.6 ± 0.4	<0.001
Body mass index (kg/m^2^)	22.8 ± 0.1	24.1 ± 0.1	24.6 ± 0.1	<0.001
Smoking				<0.001
Non-smoker	163(21.5%)	131(15.1%)	106(12.7%)	
Ex-smoker	454(49.5%)	393(46.4%)	351(39.3%)	
Current-smoker	205(29.0%)	276(38.5%)	345(48.0%)	
Alcohol drinking				<0.001
Non-drinker	375(45.7%)	238(27.1%)	138(17.5%)	
Current-drinker^1^	447(54.3%)	562(72.9%)	664(82.5%)	
Physical activity				
Inactive	677(82.4%)	684(84.3%)	683(85.1%)	0.485
Active^2^	145(17.6%)	116(15.7%)	119(14.9%)	
	**Tertile 1 (0 ~ 14 U/L)**	**Tertile 2 (15 ~ 19 U/L)**	**Tertile 3 (20 ~ 48 U/L)**	
**Women (n = 3652)**	N = 1273	N = 1101	N = 1278	
Age (years)	53.1 ± 0.4	55.6 ± 0.4	56.5 ± 0.4	<0.001
Body mass index (kg/m^2^)	22.9 ± 0.1	23.6 ± 0.1	24.7 ± 0.1	<0.001
Smoking				0.174
Non-smoker	1192(92.8%)	1019(91.9%)	1174(90.6%)	
Ex-smoker	41(3.8%)	41(3.7%)	45(3.5%)	
Current-smoker	40(3.4%)	41(4.4%)	59(5.8%)	
Alcohol drinking				0.008
Non-drinker	913(68.9%)	741(62.6%)	831(62.3%)	
Current-drinker^1^	360(31.1%)	360(37.4%)	447(37.7%)	
Physical activity				
Inactive	1079(86.1%)	964(87.9%)	1087(83.8%)	0.073
Active^2^	194(13.9%)	137(12.1%)	191(16.2%)	

All statistics are results from complex survey data analyses.

^1^participants with alcohol drinking on one or more times of the past 30 days.

^2^participants with moderate or vigorous physical activity on 5 or more days of the past 7 days.

Table 2 shows the associations of IFG with serum GGT or BMI. The risk of IFG was 2 − 3 times higher among men and women with serum GGT belonging to the 3rd tertile of the normal range after adjusting for age, smoking, alcohol intake, and physical activity. Further adjustment for BMI did not materially change the association between serum GGT and IFG. Associations between BMI and IFG were also observed in men and women. Adjusted ORs by tertile of GGT were 1.0, 2.0, and 2.7 in men and 1.0, 1.7, and 2.8 in women (P for trend < 0.001 for both genders).

**Table 2:** Prevalence and adjusted odds ratios of impaired fasting glucose by tertile of serum γ-glutamyltransferase (GGT) and category of body mass index (BMI)

	**Tertiles of serum GGT**			**P trend**
**Men**	**Tertile 1 (0 ~ 22 U/L)**	**Tertile 2 (23 ~ 34 U/L)**	**Tertile 3 (35 ~ 73 U/L)**	
Case/participants	184/822	240/800	315/802	
Prevalence, %	19.5%	28.0%	39.4%	
Odds ratio (95% CI)				
Model 1	reference	1.6(1.2-2.2)	2.8(2.1-3.8)	<0.001
Model 2	reference	1.4(1.0-1.9)	2.3(1.7-3.1)	<0.001
**Women**	**Tertile 1 (0 ~ 14 U/L)**	**Tertile 2 (15 ~ 19 U/L)**	**Tertile 3 (20 ~ 48 U/L)**	
Case/participants	151/1273	226/1101	381/1278	
Prevalence, %	10.9%	19.7%	28.8%	
Odds ratio (95% CI)				
Model 1	reference	1.9(1.4-2.5)	3.1(2.4-4.0)	<0.001
Model 2	reference	1.8(1.3-2.3)	2.5(1.9-3.3)	<0.001
	**Categories of BMI**			
	**<23 kg/m**^**2**^	**23-25 kg/m**^**2**^	**>25 kg/m**^**2**^	
**Men**				
Case/participants	215/977	217/655	307/792	
Prevalence, %	18.8%	32.5%	38.4%	
Odds ratio (95% CI)				
Model 1	reference	2.0(1.6-2.6)	2.7(2.2-3.4)	<0.001
Model 2	reference	1.9(1.5-2.4)	2.3(1.8-2.9)	<0.001
**Women**				
Case/participants	211/1570	194/938	353/1144	
Prevalence, %	12.4%	20.6%	29.3%	
Odds ratio (95% CI)				
Model 1	reference	1.7(1.3-2.3)	2.8(2.2-3.6)	<0.001
Model 2	reference	1.6(1.2-2.1)	2.4(1.8-3.1)	<0.001

All statistics, except case/participants, are results from complex survey data analyses.

Model 1: adjustment for age, smoking, alcohol intake, and physical activity.

Model 2: Further adjustment for BMI or GGT.

In Table 3, we present the associations between BMI and IFG depending on serum GGT levels in the normal range. In both men and women, BMI showed weak and statistically non-significant associations with IFG among persons with serum GGT belonging to the 1st tertile of the normal range. However, among persons with serum GGT belonging to the 2nd or 3rd tertiles of the normal range, the associations between BMI and IFG were clearly observed, with adjusted ORs ranging from 2 to 4; the P values for the multiplicative interactions were 0.032 for men and 0.059 for women. When we used waist circumference as an index of obesity, the association between waist circumference and IFG tended to become stronger as serum GGT increased, especially among women. However, the overall patterns of waist circumference were weaker than those of BMI (see Additional file 1: Table S1).

**Table 3:** **Prevalence and adjusted**^**1 **^**odds ratios of Impaired fasting glucose by category of body mass index (BMI) after stratification by tertile of serum γ-glutamyltransferase (GGT)**

	**BMI**			**P trend**	**P interaction**
	**<23 kg/m**^**2**^	**23-25 kg/m**^**2**^	**>25 kg/m**^**2**^		
**Men (n = 2424)**					0.032
**GGT tertile 1 (0 ~ 22 U/L)**					
Case/participants	95/447	45/204	44/171	0.193	
Prevalence	17.2%	21.2%	23.4%		
Adjusted OR (95% CI)	reference	1.2(0.7-2.1)	1.4(0.8-2.3)		
**GGT tertile 2 (23 ~ 34 U/L)**					
Case/participants	66/297	71/213	103/290	0.001	
Prevalence (%)	19.8%	31.4%	32.4%		
Adjusted OR (95% CI)	reference	1.9(1.2-3.1)	2.1(1.3-3.3)		
**GGT tertile 3 (35 ~ 73 U/L)**					
Case/participants	54/233	101/238	160/331	<0.001	
Prevalence (%)	20.2%	42.0%	49.5%		
Adjusted OR (95% CI)	reference	2.9(1.8-4.8)	4.0(2.5-6.4)		
**Women (n = 3652)**					0.059
**GGT tertile 1 (0 ~ 14 U/L)**					
Case/participants	73/678	35/352	43/243	0.100	
Prevalence (%)	9.8%	9.5%	15.8%		
Adjusted OR (95% CI)	reference	0.9(0.6-1.5)	1.7(1.0-2.8)		
**GGT tertile 2 (15 ~ 19 U/L)**					
Case/participants	66/491	65/262	95/348	<0.001	
Prevalence (%)	13.2%	24.1%	26.0%		
Adjusted OR (95% CI)	reference	2.1(1.2-3.5)	2.2(1.4-3.5)		
**GGT tertile 3 (20 ~ 48 U/L)**				<0.001	
Case/participants	72/401	94/324	215/553		
Prevalence (%)	16.2%	29.7%	37.4%		
Adjusted OR (95% CI)	reference	2.1(1.3-3.2)	3.1(2.1-4.5)		

All statistics, except the numbers of case/participants, are results from complex survey data analyses.

^1^Adjustment for age, smoking, alcohol intake, and physical activity.

When we repeated the same analyses with T2D as the outcome measure, the patterns became weaker, and the interaction terms failed to reach statistical significance (see Additional file 1: Table S2). However, after exclusion of known T2D, the associations between BMI and newly diagnosed T2D became stronger as serum GGT increased, similar to the results of IFG; however, the interaction terms failed to reach statistical significance possibly due to the small number of cases (see Additional file 1: Table S3). When the associations between BMI and IFG were stratified by levels of serum alanine aminotransferase (ALT) or aspartate aminotransferase (AST), interactions with obesity were not observed. BMI was strongly associated with IFG in all strata of serum ALT or AST (see Additional file 1: Table S4 and S5).

**Figure 1:**
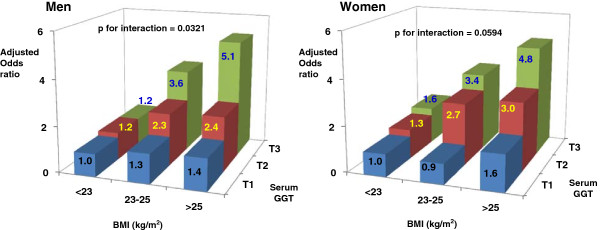
**Adjusted odds ratios by tertiles (T) of serum γ-glutamyltransferase (GGT) and category of body mass index (BMI).** BMI was classified into 3 categories (<23, 23-25, and >25 kg/m^2^) and serum GGT was categorized into gender-specific tertiles. T1: first tertile; T2: second tertile; T3: third tertile.

Figure 1 presents the results based on the common reference group of men or women with serum GGT in the lowest tertile and BMI < 23 kg/m^2^. Compared to this reference group, the risk of IFG among men with BMI ≥ 25 kg/m^2^ but serum GGT in the 1st tertile was 1.4 times higher, but men with BMI ≥ 25 kg/m^2^ and serum GGT in the 3rd tertile had about 5-fold increased risk of IFG. This pattern was observed in women as well.

## Discussion

In this study, we observed interactions between serum GGT and BMI in relation to the risk of IFG in both men and women. The associations between BMI and IFG were different depending on the serum GGT levels within the normal physiological range. Among persons belonging to the lowest tertile of serum GGT, BMI showed statistically non-significant weak associations with the risk of IFG. However, for persons within the highest tertile of serum GGT, the risk of IFG was 3 − 4 times higher among persons with BMI ≥ 25 kg/m^2^ than among those with BMI < 23 kg/m^2^. Even though serum GGT itself is reported to be related to IFG after adjusting for obesity in a dose-response relationship [15-17], to the best of our knowledge, this is the first study to evaluate whether or not the well-established association between obesity and IFG varies according to serum GGT levels.

The possibility of interactions between obesity and serum GGT associated with the risk of T2D was suggested in previous studies [3,5-10], but formal tests of multiplicative interaction terms failed to reach statistical significance in most studies [3,5,9,10]. Also, when gender-specific analyses were performed in previous studies of T2D, only women tended to show the interactions [6,8]. In fact, when we compared the results of the two outcomes of IFG and T2D in this study, the patterns were much weaker with the outcome of T2D than with that of IFG. However, when we focused on newly diagnosed T2D after excluding known T2D, the patterns became somewhat stronger than those of T2D but still weaker than those of IFG. Therefore, as we hypothesized, the interaction between obesity and serum GGT would appear in the early stages of the pathogenesis of T2D and weaken as the disease progressed.

The interactions between serum GGT and obesity in relation to the risk of IFG suggest important pathophysiological mechanisms of T2D. In particular, the weak and non-significant associations between obesity and IFG among persons with very low normal serum GGT suggest that obesity alone may be only a weak risk factor for developing IFG or T2D and that certain levels of serum GGT within the normal range may be a prerequisite condition for obesity to be strongly related to IFG or T2D. Thus, the physiological functions of GGT should be considered to interpret this finding. At least three mechanisms can be considered to explain this phenomenon: GGT as an indicator of non-alcoholic fatty liver, which is closely associated with obesity and visceral fat deposition [18]; GGT as an early marker of oxidative stress [19]; or GGT as a marker of low dose exposure to various chemical mixtures [20].

However, the possibility that serum GGT showed such interactions with obesity as a marker of non-alcoholic fatty liver may be excluded because serum ALT, a sensitive marker of non-alcoholic fatty liver [21], did not show interactions with obesity in both the current and previous study [7]. In addition, it is unlikely for serum GGT levels belonging to the 2nd and 3rd tertiles of the normal range to be related to any pathological condition in the liver.

Both experimental and clinical studies suggest that oxidative stress plays a major role in the pathogenesis of T2D and its complications [22]. Also, increased oxidative stress in accumulated fat is an important mechanism of the obesity-associated metabolic syndrome [23]. Therefore, if serum GGT within the normal range is a marker of oxidative stress [19], the interactions between serum GGT and obesity may be biologically plausible because obese persons with elevated serum GGT can be regarded as those at high risk of obesity-related diseases due to oxidative stress. However, even though there are many common biomarkers of oxidative stress in humans [24], no published studies have evaluated the interactions between these markers and obesity with regards to the risk of T2D.

Another potential mechanism is that serum GGT may be a marker of low dose exposure to chemical mixtures because cellular GGT is a necessary enzyme to metabolize glutathione conjugates of some environmental chemicals, which is also closely related to oxidative stress [20]. Interestingly, there were interactions between persistent organic pollutants (POPs), lipophilic chemical mixtures stored in adipose tissue and continuously released to circulation, and obesity in relation to the risk of T2D [25-27], parallel to the findings of serum GGT and obesity; the relationship between obesity and T2D became stronger as the serum concentrations of POPs increased. In particular, obesity was not associated with T2D among persons with very low serum concentrations of POPs in one human study [27]. Based on the physiological mechanism of the induction of cellular GGT and the empirical findings on the interactions between POPs and obesity, the claim of serum GGT as an indicator for various environmental chemicals seems to be the most plausible, and the interactions between serum GGT and obesity suggest that the presence of low dose chemical mixtures like POPs is necessary for T2D to develop, particularly in obese persons.

Regardless of the mechanisms involved in the interaction between serum GGT and obesity, this finding has an important clinical implication. In fact, individuals with similar degrees of obesity can have strikingly different risks of T2D [28]. For example, about 75–80% of obese people never develop T2D even though 80% of patients with T2D are obese [29]. Insulin resistance, a prediabetic state, varies 6-fold among obese persons [30]. Thus, information on serum GGT may be helpful to predict which obese persons are at high risk of developing T2D.

This study has certain limitations. First, since this study is a cross-sectional study, a causal relationship cannot be established. However, previous studies with the outcome of T2D, both prospective [3,5,8-10] and cross-sectional [6,7] in design, showed the same tendency of the interactions. Therefore, we expect that there is a similar pattern with the incidence of IFG. In fact, cross-sectional studies with the outcome of T2D, rather than IFG, can be more complicated to interpret because patients with T2D may try to lose weight, and serum GGT can also be affected by changes in body weight [31]. Second, there may be a misclassification bias due to the single measurement of fasting glucose or serum GGT. However, the inaccuracy of diagnosing IFG or classifying serum GGT may lead to a non-differential misclassification, which would make the true association weaker.

## Conclusion

In conclusion, considering the current and previous findings showing the different relationships of obesity with IFG and T2D according to serum GGT levels within the normal range, obesity itself may be only weakly associated with IFG and T2D when GGT levels are very low. Underlying factors that physiologically, but not pathologically, induce increased serum GGT levels, may be more critical factors in developing T2D, raising questions about the current dogma regarding the association between obesity and T2D. In addition, as the measurement of serum GGT is easy and cheap, it could be used for early detection of high-risk obese persons in the clinical field.

## Competing interests

The authors declare that they have no competing interests.

## Authors’ contributions

NSH wrote the draft. YML and HWK performed the statistical analyses. JGK, KSK, SK, and KYK contributed to discussions and edited the manuscript. DHL conceived of the study design, supervised analyses, and edited the manuscript. All authors have read and approved the final manuscript.

## Pre-publication history

The pre-publication history for this paper can be accessed here:

http://www.biomedcentral.com/1472-6823/14/57/prepub

## Supplementary Material

Additional file 1: Table S1 Prevalence and adjusted odds ratios of impaired fasting glucose by tertile of serum γ-glutamyltransferase (GGT) and tertile of waist circumference. **Table S2.** Prevalence and adjusted odds ratios of type 2 diabetes by category of body mass index (BMI) after stratification by tertile of serum γ-glutamyltransferase (GGT). **Table S3.** Prevalence and adjusted^1^ odds ratios of newly diagnosed type 2 diabetes by category of body mass index (BMI) after stratification by tertile of serum γ-glutamyltransferase (GGT). **Table S4.** Prevalence and adjusted^1^ odds ratios of Impaired fasting glucose by category of body mass index (BMI) after stratification by tertile of serum alanine aminotransferase (ALT) within normal range. **Table S5.** Prevalence and adjusted^1^ odds ratios of Impaired fasting glucose by category of body mass index (BMI) after stratification by tertile of serum asparate aminotransferase (AST) within normal range. Click here for file
