# Supplementary material for: Different associations between obesity and impaired fasting glucose depending on serum gamma-glutamyltransferase levels within normal range: a cross-sectional study
Source: BMC Endocr Disord. 2014 Jul 12;14:57. doi: 10.1186/1472-6823-14-57 (PMC4107621; doi:10.1186/1472-6823-14-57)
Supplement: Additional file 1: Table S1 — Prevalence and adjusted odds ratios of impaired fasting glucose by tertile of serum γ-glutamyltransferase (GGT) and tertile of waist circumference. Table S2. Prevalence and adjusted odds ratios of type 2 diabetes by category of body mass index (BMI) after stratification by tertile of serum γ-glutamyltransferase (GGT). Table S3. Prevalence and adjusted1 odds ratios of newly diagnosed type 2 diabetes by category of body mass index (BMI) after stratification by tertile of serum γ-glutamyltransferase (GGT). Table S4. Prevalence and adjusted1 odds ratios of Impaired fasting glucose by category of body mass index (BMI) after stratification by tertile of serum alanine aminotransferase (ALT) within normal range. Table S5. Prevalence and adjusted1 odds ratios of Impaired fasting glucose by category of body mass index (BMI) after stratification by tertile of serum asparate aminotransferase (AST) within normal range. [file 1472-6823-14-57-S1.docx]

Additional file 1: Table S1. Prevalence and adjusted odds ratios of impaired fasting glucose by tertile of serum γ-glutamyltransferase (GGT) and tertile of waist circumference

|  | **Waist circumference** | | | **P trend** | **P interaction** |  |
| --- | --- | --- | --- | --- | --- | --- |
|  | **Tertile1**  **(~81.1cm)** | **Tertile2**  **(81.2~88.0cm)** | **Tertile3**  **(88.1cm~)** |  |  |  |
| **Men (n=2419)** |  |  |  |  | 0.596 | |
| **GGT tertile1(0~22U/L)** |  |  |  |  |  |  |
| Case/participants | 66/368 | 62/268 | 54/181 | 0.045 |  |  |
| Prevalence | 15.7% | 21.0% | 26.0% |  |  |  |
| Adjusted OR(95% CI) | Reference | 1.3 (0.8-2.2) | 1.7(1.0-3.0) |  |  |  |
| **GGT tertile2(23~35U/L)** |  |  |  |  |  |  |
| Case/participants | 57/254 | 75/273 | 108/272 | 0.003 |  |  |
| Prevalence(%) | 22.4% | 24.1% | 37.1% |  |  |  |
| Adjusted OR(95% CI) | Reference | 1.1 (0.7-1.7) | 2.1 (1.3-3.4) |  |  |  |
| **GGT tertile3(36~73U/L)** |  |  |  |  |  |  |
| Case/participants | 45/179 | 96/278 | 172/346 | <0.001 |  |  |
| Prevalence(%) | 26.5% | 32.7% | 50.5% |  |  |  |
| Adjusted OR(95% CI) | Reference | 1.3 (0.8-2.2) | 2.7 (1.6-4.4) |  |  |  |
|  | **Waist circumference** | | |  |  |  |
|  | **Tertile1**  **(~75.7cm)** | **Tertile2**  **(75.8~83.3cm)** | **Tertile3**  **(83.4Cm~)** |  |  |  |
| **Women (n=3648)** |  |  |  |  | 0.097 | |
| **GGT tertile 1(0~14U/L)** |  |  |  |  |  |  |
| Case/participants | 49/544 | 57/471 | 45/255 | 0.027 |  |  |
| Prevalence(%) | 8.4% | 11.3% | 15.7% |  |  |  |
| Adjusted OR(95% CI) | Reference | 1.4 (0.8-2.3) | 1.8(1.1-3.1) |  |  |  |
| **GGT tertile2(15~20U/L)** |  |  |  |  |  |  |
| Case/participants | 41/378 | 81/358 | 102/364 | <0.001 |  |  |
| Prevalence(%) | 9.8% | 23.8% | 26.2% |  |  |  |
| Adjusted OR(95% CI) | Reference | 2.6(1.6-4.2) | 2.9(1.7-4.8) |  |  |  |
| **GGT tertile3(21~48U/L)** |  |  |  | <0.001 |  |  |
| Case/participants | 44/291 | 106/394 | 232/593 |  |  |  |
| Prevalence(%) | 14.8% | 24.4% | 39.2% |  |  |  |
| Adjusted OR(95% CI) | Reference | 1.7 (1.0-2.8) | 3.3 (2.1-5.4) |  |  |  |

All statistics, except the numbers of case/participants, are results from complex survey data analyses

^1^Adjustment for age, smoking, alcohol intake, and physical activity

Table S2.Prevalence and adjusted^1^ odds ratios of type 2 diabetes by category of body mass index(BMI) after stratification by tertile of serum γ-glutamyltransferase(GGT)

|  | **BMI** | | | **P trend** | **P interaction** |  |
| --- | --- | --- | --- | --- | --- | --- |
|  | **<23** kg/m^2^ | **23-25** kg/m^2^ | **>25**kg/m^2^ |  |  |  |
| **Men (n=2876)** |  |  |  |  | 0.924 | |
| **GGT tertile1(0~22U/L)** |  |  |  |  |  |  |
| Case/participants | 63/510 | 36/240 | 28/199 | 0.233 |  |  |
| Prevalence | 10.7% | 15.3% | 14.2% |  |  |  |
| Adjusted OR(95% CI) | reference | 1.5 (0.8-2.7) | 1.4 (0.7-2.5) |  |  |  |
| **GGT tertile2(23~35U/L)** |  |  |  |  |  |  |
| Case/participants | 47/361 | 42/267 | 50/353 | 0.114 |  |  |
| Prevalence(%) | 10.3% | 12.0% | 11.5% |  |  |  |
| Adjusted OR(95% CI) | reference | 1.4 (0.7-2.8) | 1.6 (0.9-2.8) |  |  |  |
| **GGT tertile3(36~73U/L)** |  |  |  |  |  |  |
| Case/participants | 38/254 | 55/281 | 93/411 | 0.069 |  |  |
| Prevalence(%) | 14.1% | 17.9% | 19.8% |  |  |  |
| Adjusted OR(95% CI) | reference | 1.5 (0.9-2.8) | 1.7 (1.0-3.0) |  |  |  |
| **Women (n=4126)** |  |  |  |  | 0.270 | |
| **GGT tertile 1(0~14U/L)** |  |  |  |  |  |  |
| Case/participants | 48/726 | 16/368 | 24/267 | 0.979 |  |  |
| Prevalence(%) | 5.5% | 3.6% | 6.1% |  |  |  |
| Adjusted OR(95% CI) | reference | 0.6 (0.3-1.4) | 1.1 (0.6-2.1) |  |  |  |
| **GGT tertile2(15~20U/L)** |  |  |  |  |  |  |
| Case/participants | 32/575 | 33/338 | 55/460 | 0.002 |  |  |
| Prevalence(%) | 5.0% | 7.2% | 12.5% |  |  |  |
| Adjusted OR(95% CI) | reference | 1.4 (0.7-2.6) | 2.8 (1.5-5.4) |  |  |  |
| **GGT tertile3(21~48U/L)** |  |  |  | 0.004 |  |  |
| Case/participants | 52/401 | 56/337 | 158/654 |  |  |  |
| Prevalence(%) | 13.5% | 16.0% | 21.7% |  |  |  |
| Adjusted OR(95% CI) | reference | 1.1 (0.6-1.8) | 1.7 (1.1-2.5) |  |  |  |

All statistics, except the numbers of case/participants, are results from complex survey data analyses

^1^Adjustment for age, smoking, alcohol intake, and physical activity

Table S3. Prevalence and adjusted^1^ odds ratios of newly diagnosed type 2 diabetes by category of body mass index(BMI) after stratification by tertile of serum γ-glutamyltransferase(GGT)

|  | **BMI** | | | **P trend** | **P interaction** |  |
| --- | --- | --- | --- | --- | --- | --- |
|  | **<23** kg/m^2^ | **23-25** kg/m^2^ | **>25**kg/m^2^ |  |  |  |
| **Men (n=2502)** |  |  |  |  | 0.283 | |
| **GGT tertile1(0~22U/L)** |  |  |  |  |  |  |
| Case/participants | 7/454 | 8/212 | 1/172 | 0.375 |  |  |
| Prevalence | 1.6% | 2.9% | 0.5% |  |  |  |
| Adjusted OR(95% CI) | reference | 1.7 (0.5-6.0) | 0.3(0.0-2.1) |  |  |  |
| **GGT tertile2(23~34U/L)** |  |  |  |  |  |  |
| Case/participants | 4/301 | 3/216 | 9/299 | 0.106 |  |  |
| Prevalence(%) | 0.8% | 2.5% | 2.7% |  |  |  |
| Adjusted OR(95% CI) | reference | 3.3 (0.8-14.4) | 3.6 (0.9-14.1) |  |  |  |
| **GGT tertile3(36~73U/L)** |  |  |  |  |  |  |
| Case/participants | 7/240 | 16/254 | 23/354 | 0.052 |  |  |
| Prevalence(%) | 2.1% | 7.7% | 7.3% |  |  |  |
| Adjusted OR(95% CI) | reference | 4.2 (1.5-11.9) | 3.3 (1.2-8.9) |  |  |  |
| **Women (n=3724)** |  |  |  |  | 0.463 | |
| **GGT tertile 1(0~14U/L)** |  |  |  |  |  |  |
| Case/participants | 3/681 | 1/353 | 2/245 | 0.495 |  |  |
| Prevalence(%) | 0.3% | 0.1% | 0.7% |  |  |  |
| Adjusted OR(95% CI) | reference | 0.5 (0.0-4.8) | 2.3 (0.4-15.2) |  |  |  |
| **GGT tertile2(15~20U/L)** |  |  |  |  |  |  |
| Case/participants | 2/545 | 2/307 | 9/414 | 0.098 |  |  |
| Prevalence(%) | 0.5% | 0.2% | 2.2% |  |  |  |
| Adjusted OR(95% CI) | reference | 0.4 (0.0-3.7) | 4.2(0.8-21.2) |  |  |  |
| **GGT tertile3(21~48U/L)** |  |  |  | 0.007 |  |  |
| Case/participants | 7/356 | 10/291 | 36/532 |  |  |  |
| Prevalence(%) | 2.2% | 4.4% | 7.2% |  |  |  |
| Adjusted OR(95% CI) | reference | 1.9 (0.7-5.8) | 3.3 (1.3-8.3) |  |  |  |

All statistics, except the numbers of case/participants, are results from complex survey data analyses

^1^Adjustment for age, smoking, alcohol intake, and physical activity

Table S4. Prevalence and adjusted^1^ odds ratios of Impaired fasting glucose by category of body mass index(BMI) after stratification by tertile of serum alanine aminotransferase (ALT) within normal range

|  | **BMI** | | | **P trend** | **P interaction** |  |
| --- | --- | --- | --- | --- | --- | --- |
|  | **<23** kg/m^2^ | **23-25** kg/m^2^ | **>25**kg/m^2^ |  |  |  |
| **Men (n=2570)** |  |  |  |  | 0.244 | |
| **ALT tertile 1(0~16U/L)** |  |  |  |  |  |  |
| Case/participants | 108/490 | 68/205 | 54/184 | 0.001 |  |  |
| Prevalence | 17.4% | 32.5% | 29.1% |  |  |  |
| Adjusted OR (95% CI) | reference | 2.4 (1.6-3.7) | 2.0 (1.2-3.2) |  |  |  |
| **ALT tertile2(17~22U/L)** |  |  |  |  |  |  |
| Case/participants | 71/312 | 68/233 | 88/241 | 0.039 |  |  |
| Prevalence(%) | 22.6% | 26.4% | 32.3% |  |  |  |
| Adjusted OR (95% CI) | reference | 1.2 (0.7-2.0) | 1.6 (1.0-2.5) |  |  |  |
| **ALT tertile 3(23~40U/L)** |  |  |  |  |  |  |
| Case/participants | 59/239 | 113/273 | 176/393 | <0.001 |  |  |
| Prevalence(%) | 21.1% | 38.0% | 42.8% |  |  |  |
| Adjusted OR (95% CI) | reference | 2.4 (1.5-3.7) | 2.9 (1.9-4.5) |  |  |  |
| **Women (n=3710)** |  |  |  |  | 0.671 | |
| **ALT tertile 1(0~13U/L)** |  |  |  |  |  |  |
| Case/participants | 77/715 | 52/324 | 65/239 | <0.001 |  |  |
| Prevalence(%) | 10.4% | 15.4% | 26.6% |  |  |  |
| Adjusted OR (95% CI) | Reference | 1.5 (0.9-2.5) | 2.9 (1.8-4.6) |  |  |  |
| **ALT tertile2(14~17U/L)** |  |  |  |  |  |  |
| Case/participants | 78/495 | 56/302 | 95/330 | <0.001 |  |  |
| Prevalence(%) | 13.4% | 17.6% | 27.6% |  |  |  |
| Adjusted OR(95% CI) | reference | 1.3 (0.9-2.1) | 2.5 (1.7-3.7) |  |  |  |
| **ALT tertile 3(18~40U/L)** |  |  |  | <0.001 |  |  |
| Case/participants | 64/393 | 89/327 | 198/585 |  |  |  |
| Prevalence(%) | 16.6% | 29.1% | 32.0% |  |  |  |
| Adjusted OR(95% CI) | reference | 2.0 (1.2-3.2) | 2.3 (1.5-3.6) |  |  |  |

All statistics, except the numbers of case/participants, are results from complex survey data analyses

^1^Adjustment for age, smoking, alcohol intake, and physical activity

Table S5. Prevalence and adjusted^1^ odds ratios of Impaired fasting glucose by category of body mass index(BMI) after stratification by tertile of serum asparate aminotransferase(AST) within normal range

|  | **BMI** | | | **P trend** | **P interaction** |  |
| --- | --- | --- | --- | --- | --- | --- |
|  | **<23** kg/m^2^ | **23-25** kg/m^2^ | **>25**kg/m^2^ |  |  |  |
| **Men (n=2680)** |  |  |  |  | 0.859 | |
| **AST tertile1(0~19U/L)** |  |  |  |  |  |  |
| Case/participants | 69/354 | 65/216 | 71/219 | <0.001 |  |  |
| Prevalence | 15.1% | 25.8% | 31.3% |  |  |  |
| Adjusted OR(95% CI) | reference | 1.9 (1.2-3.1) | 2.7 (1.7-4.1) |  |  |  |
| **AST tertile2(20~24U/L)** |  |  |  |  |  |  |
| Case/participants | 88/356 | 105/303 | 137/351 | 0.002 |  |  |
| Prevalence(%) | 23.8% | 34.4% | 39.6% |  |  |  |
| Adjusted OR (95% CI) | reference | 1.6 (1.0-2.4) | 1.9 (1.3-3.0) |  |  |  |
| **AST tertile3(25~40U/L)** |  |  |  |  |  |  |
| Case/participants | 84/324 | 88/219 | 151/338 | <0.001 |  |  |
| Prevalence(%) | 21.9% | 37.6% | 42.7% |  |  |  |
| Adjusted OR(95% CI) | reference | 2.3 (1.5-3.5) | 2.8 (1.8-4.3) |  |  |  |
| **Women (n=3756)** |  |  |  |  | 0.773 | |
| **AST tertile1(0~18U/L)** |  |  |  |  |  |  |
| Case/participants | 83/662 | 71/377 | 110/367 | <0.001 |  |  |
| Prevalence(%) | 11.8% | 17.9% | 28.2% |  |  |  |
| Adjusted OR(95% CI) | reference | 1.5 (0.9-2.3) | 2.6 (1.7-4.0) |  |  |  |
| **AST tertile2(19~21U/L)** |  |  |  |  |  |  |
| Case/participants | 53/436 | 44/252 | 89/320 | <0.001 |  |  |
| Prevalence(%) | 10.4% | 16.7% | 26.0% |  |  |  |
| Adjusted OR (95% CI) | reference | 1.7 (1.0-2.8) | 2.9 (1.8-4.7) |  |  |  |
| **AST tertile3(22~40U/L)** |  |  |  | <0.001 |  |  |
| Case/participants | 85/515 | 87/338 | 160/489 |  |  |  |
| Prevalence(%) | 16.5% | 27.8% | 31.5% |  |  |  |
| Adjusted OR (95% CI) | reference | 2.0 (1.3-2.9) | 2.4 (1.7-3.5) |  |  |  |

All statistics, except the numbers of case/participants, are results from complex survey data analyses

^1^Adjustment for age, smoking, alcohol intake, and physical activity
